# Let There Be Light!

**DOI:** 10.3390/proteomes4040036

**Published:** 2016-11-29

**Authors:** Doroteya Raykova, Björn Koos, Anna Asplund, Márton Gelléri, Ylva Ivarsson, U. Helena Danielson, Ola Söderberg

**Affiliations:** 1 Department of Pharmaceutical Biosciences, Pharmaceutical Cell Biology, Biomedical Center, Box 594, Uppsala University, SE-751 08 Uppsala, Sweden; doroteya.raykova@igp.uu.se; 2 Department of Systemic Cell Biology, Max Planck Institute of Molecular Physiology, Otto-Hahn-Straße 11, 44227 Dortmund, Germany; bjoern.koos@mpi-dortmund.mpg.de (B.K.); marton.gelleri@mpi-dortmund.mpg.de (M.G.); 3 Department of Immunology, Genetics and Pathology, Dag Hamarskjölds väg 14B, Rudbeck Laboratory, Uppsala University, 751 85 Uppsala, Sweden; anna.asplund@igp.uu.se; 4 Faculty of Chemistry and Chemical Biology, Technical University of Dortmund, Otto-Hahn-Straße 6, 44221 Dortmund, Germany; 5 Department of Chemistry-BMC, Box 576, Uppsala University, SE-751 23 Uppsala, Sweden; ylva.ivarsson@kemi.uu.se (Y.I.); helena.danielson@kemi.uu.se (U.H.D.)

**Keywords:** high resolution microscopy, protein–protein interactions, post-translational modifications, FRET, *in situ* PLA, proxHCR

## Abstract

The invention of the microscope has been fundamental for the understanding of tissue architecture and subcellular structures. With the advancement of higher magnification microscopes came the development of various molecular biology tools such as Förster resonance energy transfer (FRET) and *in situ* proximity ligation assay (*in situ* PLA) to monitor protein interactions. Microscopy has become a commonly used method for the investigation of molecular events within the cell, for the identification of key players in signaling networks, and the activation of these pathways. Multiple approaches are available for functional analyses in single cells. They provide information not only on the localization of proteins at a given time point, but also on their expression levels and activity states, allowing us to pinpoint hallmarks of different cellular identities within tissues in health and disease. Clever solutions to increase the sensitivity of molecular tools, the possibilities for multiplexing, as well as image resolution have recently been introduced; however, these methods have their pros and cons. Therefore, one needs to carefully consider the biological question of interest along with the nature of the sample before choosing the most suitable method or combination of methods. Herein, we review a few of the most exciting microscopy-based molecular techniques for proteomic analysis and cover the benefits as well as the disadvantages of their use.

## 1. Introduction

Vision is the prime sensory input humans use in order to relate to the world, and much of our knowledge is based on accumulated interpretations of visual information and experiences. In order to observe and study faraway objects, devices that enhance our capabilities were developed. Telescopes enabled us to see further, giving us insights into the nature of our world and giving rise to whole new branches of science: from aiding navigation, to the development of astrophysics. In order to understand not only the surrounding world, but also ourselves as part of it, early scientists conducted anatomic studies which revealed that the body is built up of different organs, each with its own function. However, the building blocks comprising these organs remained unknown for a long time, since observing extremely small objects turned out to be equally challenging as seeing at a distance. Thus, the development of the microscope opened a fantastic new microcosm for us to explore, and—in a biological context—revealed the composition of tissues and the architecture of cells. Proteins emerged as macromolecules with a pivotal role within the cell, such as performing structural functions, responding to internal and environmental cues, and catalyzing different biochemical reactions that allow the survival and renewal of the organism. Therefore, understanding protein function and composition (which is largely related to function as well) is an essential task which requires a variety of sensitive and reliable methods.

However, it is important to not only detect the presence/absence of a protein, but also to determine its functional states; i.e., whether it carries post-translational modifications or is present in complex with other proteins, in which cells it is expressed, and in which cell compartments the different states of the protein are present. The challenges of the proteomics field thus include understanding and discovering all these different aspects in an adequate and comprehensive way. Mass spectrometry provides the ability to determine the potential presence of thousands of different proteins in a sample, and with targeted approaches, their levels as well, which will contribute to revolutionizing the field of proteomics in a way similar to how next generation sequencing has done in the field of genomics. Although mass spectrometry can be used to detect proteins in tissue sections [1,2,3], microscopy-based techniques such as immunohistochemistry/immunofluorescence remain indispensable tools to verify and add information to large-scale proteomic studies, providing evidence on where a protein is expressed at a cellular and even sub-cellular level [4,5].

Three important things to consider in microscopy are the resolution of the method, the ability to specifically target a particular protein, and the strength of the generated signal. On a biological level one should also consider how much the system is disturbed by the experimental setup of the assay that is performed. Dyes and fluorescent molecules have been instrumental for visualization of cells and subcellular components that do not emit light per se. To target the chromophores and fluorophores to a specific protein, antibodies and other affinity reagents can be used. Alternatively, the protein of interest can be fused to a fluorescent protein, such as the green fluorescent protein (GFP) or citrine, so that it can be observed directly in live or fixed cells [6]. Analysis of physical interactions between proteins to determine levels of protein complex formation requires other methods, such as co-immunoprecipitation, but there are also several microscopy-based methods that retain the spatial information on where in the cells and sub-cellular compartments a protein–protein interaction occurs. We herein describe a few of these methods, together with their caveats and advantages, which may be useful for validating and further characterizing the findings from mass spectrometry.

## 2. I Can See Clearly Now, the Rain Is Gone

The German physicist Ernst Karl Abbe discovered that the smallest structures that can be resolved by a light microscope are approximately half the wavelength of the light detected, divided by the numerical aperture of the objective. This fundamental limit is called the diffraction limit. In practical terms, this means that for green light with a wavelength of 500 nm, the smallest objects that can be resolved are around 250 nm in size. This is sufficient for many biological applications, since a cell is several micrometers in diameter. However, the size of cellular organelles and proteins fall below the diffraction barrier of visible light.

In 2014, the Nobel Prize in chemistry was awarded to Stefan Hell, Eric Betzig and William Moerner for circumventing the limit in far field fluorescence microscopy. Their inventions use the temporal and spatial modulation of light to increase resolution beyond the diffraction limit. Stimulated emission depletion (STED) microscopy is a laser scanning microscopy method in which a donut-shaped beam is overlaid onto the excitation beam [7,8]. This beam de-excites fluorophores in the excited state by stimulated emission. The intensity of the donut-shaped beam is zero in its very center; therefore only fluorophores slightly off-center of the excitation beam will be depleted and are de-excited back to the ground state. This leads to an effective reduction of the volume in which a fluorophore can be excited. Resolutions below 50 nm are routinely achieved with STED microscopy [9,10]. Localization-based microscopy methods such as photoactivated localization microscopy (PALM) and stochastic optical reconstruction microscopy (STORM) are essentially widefield single molecule imaging techniques. They use the stochastic switching of initially dark fluorophores to a fluorescent state to reduce the number of simultaneously fluorescing molecules [11,12]. By switching only a small subset of molecules to the fluorescent state, the mean distance between fluorescing molecules in an image becomes much larger than the diffraction limit of light. This allows the collected signal to be assigned to individual molecules and the recorded intensity distribution of each molecule to be fitted. The precision by which the center of a single molecule can be determined depends only on the signal-to-noise ratio, and is smaller than the actual resolution limit. To generate a super-resolution image, a sequence of images is recorded. In each image, only a small subset of the molecules is switched to the fluorescent state, with optimally one molecule at a time emitting light in a diffraction-limited region. The positions of all molecules are combined to a super-resolution image. These diffraction barrier-breaking inventions were brilliant, and the images produced have a fantastic sharpness compared to confocal microscopy, pushing the resolution down to less than 100 nm.

**Figure 1:**
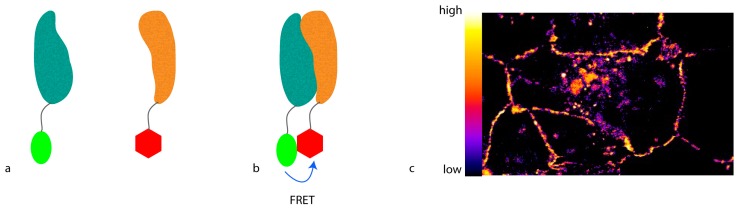
Förster resonance energy transfer (FRET). (**a**) Fluorescent proteins (proteins in teal and ochre; donor molecule in green, and acceptor in red) fused to the protein of interest are expressed ectopically in the cell. In the absence of interaction between the proteins, no energy transfer occurs; (**b**) If interaction occurs, the two fluorescent proteins come in close proximity to each other. FRET can occur due to energy transfer from the donor to the acceptor molecule; (**c**) Depending on the readout, a heat-map can be generated, showing regions of high and low interaction.

To develop light microscopy to become a truly molecular technique for proteomic studies with which not only protein localization can be visualized, but also protein interactions or conformational changes can be monitored, the resolution has to be further increased. To achieve this kind of resolution, molecular methods that involve a distance requirement can be used. A feature of such a method can be that the proximity between two molecules alters a signal only if the molecules are closer than a defined threshold. This can be obtained by Förster resonance energy transfer (FRET) (Figure 1), where energy is transferred from an excited donor fluorophore to an acceptor fluorophore that emits light at a longer wavelength. For this purpose, the emission spectrum of the donor fluorophore has to at least partly overlap with the excitation spectrum of the acceptor fluorophore. Moreover, the donor and the acceptor fluorophores have to be closer than 10 nm, defined by the so-called R0 radius that largely depends on the fluorophore pair used. In addition, the spatial orientation between the fluorophores has to favor energy transfer.

The amount of FRET can be recorded in multiple ways. The most straightforward approach is to excite the donor fluorophore and detect emission of the acceptor fluorophore. While this so-called “sensitized emission” may be the easiest way to measure FRET, it is also the most error-prone. Due to the partial overlap of the spectra, very often either the donor fluorescence can be observed in the acceptor spectrum, or the acceptor can be excited in the donor spectrum. Therefore, carefully-picked fluorophore spectra with as little overlap as possible are needed for this readout.

Alternatively, the drop in donor fluorescence can be observed. If FRET occurs, part of the energy of the excited donor will be transferred to the acceptor, and will not be used to emit light in the donor spectrum. Therefore, the donor will be dimmer than in the absence of acceptor, thus apparently quenching the signal. However, for this ratiometric imaging, the strength of the donor fluorescence in the absence of the acceptor in a certain position needs to be known. Thus, acceptor photo-bleaching is usually used when visualizing this phenomenon. Here, a picture of the sample is taken, and then the acceptor is bleached [13]. After bleaching, a second picture of the donor fluorophore is taken and the increase in fluorescence is quantified. Unfortunately, due to spectral overlap, bleaching the acceptor also in part bleaches the donor, which decreases the observed difference in fluorescence.

Lastly, the lifetime of the donor fluorophore can be measured [14]. The time the donor stays in the excited state is a physical property of the fluorophore. Since FRET provides an alternative way to reach the ground state, the average lifetime of the donor drops in the presence of an acceptor. This drop can be measured and quantified using advanced microscopes with pulsed lasers.

Another approach is to let the proximity between two molecules constitute to the requirements for the formation of a reporter molecule, which is used in methods such as protein fragment complementation. By genetically splitting an enzyme into two parts and fusing each fragment to a protein of interest, the two enzyme fragments can be brought together and thus reconstitute a functional enzyme, but only if the two proteins bind together in an appropriate orientation. An analogous example of such a method is the bimolecular fluorescence complementation assay (BiFC), where a fluorophore is split into two non-fluorescent fragments [15]. The affinity between these fragments has to be so low that the reconstitution of a functional fluorophore/enzyme requires interactions between the fusion partners.

**Figure 2:**
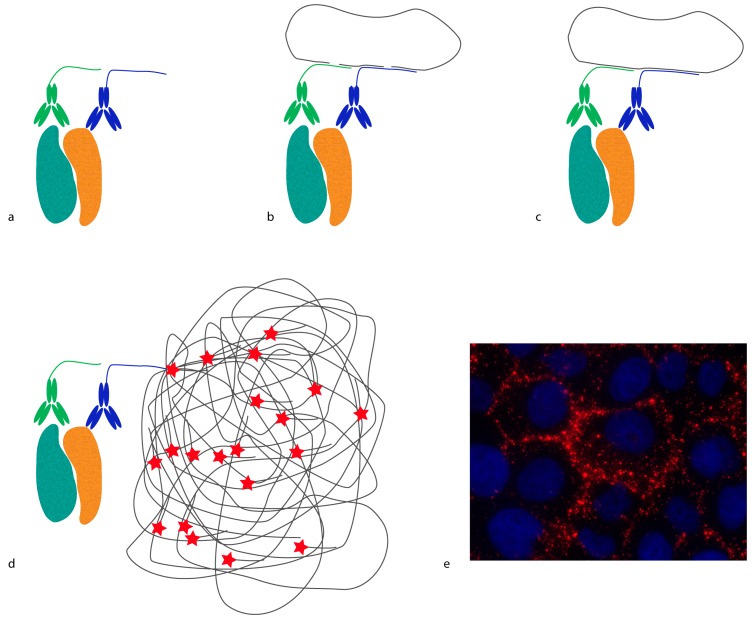
*In situ* proximity ligation assay (*in situ* PLA). (**a**) Recognition: A pair of antibodies conjugated to oligonucleotides (proximity probes) binds a protein complex (teal and ochre); (**b**) Hybridization: two circularization oligonucleotides (grey lines) with regions complementary to parts of the proximity probes’ oligonucleotide sequences are added; (**c**) Ligation: The circularization oligonucleotides are joined by ligation to form a complete DNA circle; (**d**) Rolling circle amplification (RCA): The ligated circle serves as amplification template where one of the proximity probes acts as a primer for phi29 polymerase. As a result, an RCA product is synthesized: a single-stranded DNA molecule attached to the proximity probe that contains hundreds of repeated sequences required for detection. Detection is achieved by the addition of a complementary detection oligonucleotide labeled with a fluorophore (red star); (**e**) *In situ* PLA image depicting the interaction between E-cadherin and β-catenin in MCF-10 cells. The *in situ* PLA signals appear as discrete red dots in the areas of cell-to-cell contact, and the nucleus is visualized by Hoechst staining (blue).

*In situ* proximity ligation assay (*in situ* PLA) is another example of a method that requires proximity between molecules in order to generate a reporter molecule (Figure 2). In this case, a DNA molecule is created, and it acts as a reporter of proximity [16,17]. In *in situ* PLA, short oligonucleotides coupled to antibodies (proximity probes) are brought in proximity upon binding, and act as templates/substrates for the ligation of two additional oligonucleotides into a circular molecule. The distance requirement in *in situ* PLA is determined by the length and orientation of the oligonucleotides of the proximity probes. Bearing in mind that every DNA base is 0.34 nm, a 20 base-long DNA strand is 6.8 nm. Hence, the distance threshold for reporting an interaction is less than 10 nm, counting from the point where the oligonucleotides are conjugated to the antibodies. This distance can be reduced or extended by changing the length and orientation of the oligonucleotides [18]. The ligated oligonucleotide circle can then be amplified by rolling circle amplification (RCA) using one of the oligonucleotides on the proximity probes as a primer. The amplification product is a single-stranded DNA concatemer, consisting of hundreds of repeats complementary to the circle, attached to one of the proximity probes. These can be visualized by hybridization of fluorophore-labeled oligonucleotides complementary to the RCA product. As each RCA product contains hundreds of fluorophores, they are easily detected by standard epifluorescence microscopy. The size of the RCA product is around 1 μm in diameter, and is thus much larger than the distance required to generate the DNA circle.

**Figure 3:**
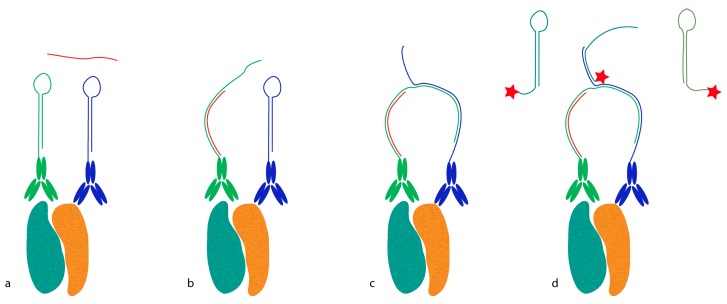

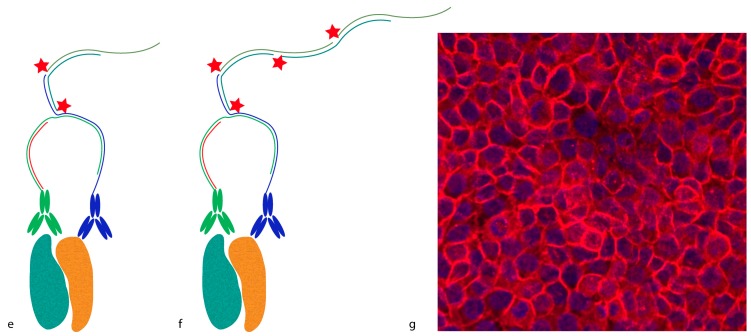
Proximity-dependent initiation of hybridization chain reaction (proxHCR). (**a**) Two proximity probes—i.e., antibodies conjugated to proximity hairpins (PH1 in green and PH2 in blue)—bind a protein complex (teal and ochre). Subsequently, an activator oligonucleotide is added (red line); (**b**) The activator breaks the secondary structure of PH1 open and binds to it, releasing its 3′-end; (**c**) The open PH1 can now invade PH2, thereby liberating its 3′-end. The latter can initiate the hybridization chain reaction (HCR); (**d**) The 3′ initiator sequence invades a fluorophore-labelled hairpin (teal line with a red star); (**e**) which then commences a chain reaction of invading and activating other such hairpins; (**f**) The reaction continues until depletion of the available fluorescent hairpins; (**g**) ProxHCR microscopy image: detection of interaction between E-cadherin and β-catenin in HaCaT cells. ProxHCR signal is shown in red and nuclei are stained in blue with Hoechst.

Proximity probes can also be constructed using oligonucleotides locked in a hairpin conformation (Figure 3) [19]. These proximity hairpins (PH) are partly reversely complementary to each other, and can cross-hybridize if they are not kinetically trapped by their secondary structure. Addition of an activator oligonucleotide that perturbs this kinetic hindrance can, upon proximal binding, lead to the opening of one PH, which in turn invades and unfolds the other PH. This liberates the 3′ end of the second PH, which was hidden in the stem before unfolding. This initiates a hybridization chain reaction (HCR), in which a double-stranded nicked DNA molecule is built up by a pair of fluorophore-labeled DNA hairpins (proxHCR). HCR is solely based on the hybridization of hairpins that are kinetically trapped as monomers in the absence of the initiator [20]. Therefore, no enzymatic steps are needed, which makes this technique more efficient and less expensive than *in situ* PLA. However, the sensitivity may be lower due to the reduced amplification capacity of proxHCR.

## 3. I Can See All Obstacles in My Way

For more than half a century, antibodies have been the primary reagents for affinity-based proteomics. The immune system displays a vast capacity to generate antibodies targeting any foreign agent through recombination in concert with somatic hypermutations. By injecting an antigen into a host animal and collecting the antibodies produced to battle the foreign substance, we can take advantage of the immune response as a fairly straightforward means to develop an affinity reagent towards any protein of interest. However, stringent targeting is not as trivial as it may sound, as there is always concern regarding selectivity, cross-reactivity, and overall quality of the affinity reagents used. While gene recombination along with somatic hypermutations generates the specificity of an antibody, highly-specific antibodies can be produced synthetically through phage display. This approach eliminates the need for the use of animals for the generation of antibodies, and is likely to become more common, as the technique is scalable and robust, and recombinant antibodies can be produced indefinitely [21,22,23].

Only a small number of amino acids (as few as 4–12) in an antibody bind the epitope of an antigen [24]. The amino acids interact via hydrogen- and ionic bonds, van der Waals interactions, and hydrophobic effects. The stability of the complex is determined by the rate at which the antibody dissociates from a protein (i.e., the off-rate). The affinity and kinetics are dependent on a number of factors, including temperature, pH, and the ionic strength of the milieu. The valency (i.e., the number of the antigen-binging sites) affects the antibody affinity through avidity effects, where high avidity speaks for a more stable antibody–antigen complex. For example, IgG has two antigen-binding sites, and thus engages in bivalent interactions; while a pentameric IgM has ten binding sites, which may result in more stable interactions, even if the affinity of each binding site for a given epitope is lower.

At equilibrium, there is a balance between the formation of an antibody–antigen complex (*Ab*–*Ag*) and its dissociation into unbound antibody (*Ab*) and antigen (*Ag*).

(1) Ab+Ag⇄Ab−Ag

The following equations reflect the relationship between the association and dissociation rates of an antibody and an antigen. The concentration of the [*Ab*–*Ag*] complex is dependent on the concentrations of unbound antibody [*Ab*], unbound antigen [*Ag*], and on the dissociation constant *K_D_*.

(2) $$K_{D}=\;\frac{k_{off}}{kon}\;\mathrm{and}\;K_{D}=\frac{\left[Ab\right]\left[Ag\right]}{\left[Ab-Ag\right]}$$

The equilibrium dissociation constant (*K_D_*) is defined as the ratio between the rate constants for dissociation and association (*k_on_* and *k_off_*, respectively), as in Equation (2). It is directly proportional to the difference in Gibbs free energy between the free and bound states (Equation (3)). The affinity is inversely proportional to *K_D_*.

(3) $$\Delta G=-RTlnK_{D}$$

These equations reflect basic biochemistry laws, but it is worth mentioning them again. It is important to note that an antibody always interacts with other proteins in addition to the intended one, albeit with different affinities. The extent of such unintended binding depends on the concentrations of the antibody and the different proteins, as well as their respective affinities.

Polyclonal antibodies recognize multiple epitopes and are therefore in theory more likely to be cross-reactive. The consequence of cross-reactivity is dependent on the difference in concentration and dissociation rate between the intended target and all other possible interactions. Setting up an assay for a highly expressed protein, where all other interactions only constitute a minority of possible events, is easy. For less abundant proteins, achieving selectivity is a considerable challenge. For such targets, it is particularly important to test the antibody for off-target effects [25].

A way to increase selectivity is to require that more than one antibody binds to the protein of interest, since the likelihood that two antibodies cross-react with the same off-target protein is much lower than their likelihood of binding to the intended target. As discussed above, only a fraction of the protein present will be bound by antibodies, and both free proteins and antibodies will be present. Thus, for an assay requiring dual binding, the fraction of the protein bound by both antibodies is even smaller. By requiring dual binding, detection becomes more selective, as it requires that both antibodies have bound the same target protein. However, as antigen binding is an equilibrium reaction, there will always be unbound targeted proteins, as well as proteins that are bound by a single antibody only, which leads to a somewhat decreased detection efficiency.

The nature and availability of antigens is another concern when working with antibodies and fixed cells or tissue sections. Antibodies can be generated to target linear epitopes; i.e., the primary structure of a stretch of amino acids, or conformational epitopes where the folding of a protein will bring distal amino acids into a secondary/tertiary structure. Consequently, it is important to consider the application when selecting the type of antibody to be used. Fixation of cells and tissue sections can denature the protein and destroy antigenic epitopes only present in folded proteins or make them unavailable to the antibody by crosslinking amino acids. The Human Protein Atlas, for example, was assembled by using antibodies raised against partially or fully denatured proteins, or recombinant protein epitopes comprising 100–150 amino acid stretches [4,5,26]. Applying these Western blot-validated antibodies for immunohistochemistry on formalin-fixed tissues and cells is not always successful [27,28]. The most likely explanation for this is that the epitope conformation differs between assays, depending on pre-treatment protocols. Therefore, users and antibody vendors alike need to recognize the fact that an antibody has to be validated for the intended application in order to avoid wasting time and resources on the generation of faulty data.

To add additional complexity, a natively folded protein can have multiple conformations, and antigenic epitopes might thus only be exposed in a fraction of the population of molecules. Changes in folding are commonly the result of interactions with other proteins or the addition of post-translational modifications (PTMs; e.g., phosphorylations), which often expose catalytic domains. This is the foundation of cellular signaling, where proteins have different activity states, and a progression of signals occurs through networks of protein–protein interactions that change these activity states. If the epitope is affected by such conformational changes, only one particular activity state may be targeted by the used antibody. Finally, an epitope may be shielded if it is present at the interface where a protein is interacting with another protein. All of these phenomena can be a reason for reduced efficiency of detection.

In summary, care should be taken before fully trusting results obtained with poorly characterized antibodies, as failure to detect a given target does not necessarily imply that the protein of interest is not there. Conversely, the signal observed when using an antibody targeting a certain protein does not guarantee that the protein is actually there.

In addition to the selection of appropriate antibodies, considering the characteristics of the sample is crucial for all methods relying on affinity reagents. Fixation, for instance, is a severely underestimated issue when it comes to protein analysis. It typically takes between 5 and 15 min for the cytoplasm of a sample to be fixed. This time span is longer when membrane-bound proteins are involved [29]. It is typically not sufficient for the degradation of expressed proteins, but enough and spare for de-phosphorylation or breakdown of spatial organization. In addition, cells show a massive toxic reaction when exposed to formaldehyde—a widely used fixative. These two features combined raise the question of whether we can actually observe the cell in its natural form when we image it after fixation. Live cell imaging solves this problem in part, because the cell can be observed in its natural environment while it is still alive. However, studying protein interactions in live cells is challenging. Although it can be achieved by transfection of fluorescent fusion proteins into the cell and monitoring (like in FRET, for example, as described above), there are many aspects to consider. Simply expressing a protein in fusion with a partner required for detection means that the protein of interest is present in the cell at a higher concentration than it usually would be, and in a non-native form. Taking into account that the occurrence of interactions is a function of protein concentration (as mentioned above, all proteins interact with each other given a high enough concentration), it seems logical that over-expressing the protein of interest may not only introduce artifacts, but can also severely change the network topology of the cell.

Additionally, FRET approaches are highly prone to false negative results. This becomes conspicuous when we take into consideration that protein interactions or PTMs often severely change the conformation of the protein of interest. So, estimating whether two domains are close enough to each other for FRET to occur can be tricky. Therefore, using a combination of methods to study the protein interaction of interest is always a good approach.

## 4. Gone Are the Dark Clouds that Had Me Blind

Although state-of-the-art microscopy can detect single fluorophores (which is the basis for techniques such as PALM and STORM), resorting to it is not trivial for samples with high autofluorescence. Just like it is difficult to see stars in daytime, the autofluorescence of a tissue section can mask the specific signal and make it difficult to observe the fluorophores. Autofluorescence is largely generated by the fixation protocol. Therefore, it does not affect live cell imaging as much. A major advantage of live cell imaging is that it allows the observation of interaction kinetics. How does the signal change over time within the same cell? A major factor in answering this question is of course photo-toxicity. Low signal-to-noise ratios (which require longer imaging time) therefore directly affect the viability of the cell, and with it the signaling networks we are trying to study. In order to counteract this, a variety of techniques have been developed. The emergence of light sheet microscopy, for example, allows the illumination of only the confocal plane, which significantly reduces photo-toxicity. Another approach is to use low temperatures. By freezing the cell to around −40 °C for imaging and thereafter warming it up to 37 °C, it is possible to reduce photo-toxicity in the cell and use long exposure times [30].

In a fixed sample, the signal-to-noise ratio improves if a detected protein or a protein interaction is visualized by multiple fluorophores in a small volume. This effect is employed in *in situ* PLA, where the signal from a single recognition event is amplified by RCA, producing an RCA product labeled with hundreds of fluorophores, which makes it stand out from the background autofluorescence. In regular epifluorescence or confocal microscopy, the RCA products are seen as discrete bright dots with a diameter of around 1 μm. When observing the RCA products with super-resolution microscopy, they are not homogenous, but consist of foci with higher fluorescence. This reflects the structure of the RCA products, where parts of the RCA product fold into a random coil conformation due to the polarity inherent to DNA, and these coils are bridged by stretches of single-stranded DNA. By introducing an additional oligonucleotide built up by repetitive elements complementary to the RCA product, distal parts are brought together, reducing the diameter of RCA products to around 200 nm [31]. Despite this, the dynamic range of *in situ* PLA remains limited to the detection of a few hundred molecular events per cell. At higher numbers, the RCA products will start to coalesce, making it impossible to discern and quantify discrete signals. To overcome this, one can use circularization oligonucleotides carrying different tags for subsequent hybridization of tag-specific oligonucleotides. These tag-specific fluorophores are labeled with unique fluorophores [32]. The ratio between the differently-colored RCA products reflects the ratio between the different tags used in the circularization oligonucleotides. Thus, it is possible to tune the dynamic range to fit a huge variation between cells.

The use of multiple fluorophore-labeled detection oligonucleotides (DOs) can also be implemented to visualize several different proteins or protein interactions in parallel [33]. By including the specific tags in the oligonucleotides of the proximity probes, the same set of circularization oligonucleotides can be used for the detection of multiple proximity events, all coded by the tag sequence carried by the proximity probes. The fluorescence of the RCA product will tell which pair of proximity probes have generated the circular ligation product. The number of discrete fluorophores that can be identified is the bottleneck for the level of multiplexing. Fluorophores with overlapping excitation and emission spectra can be difficult to separate. To further increase the level of multiplexing, a few different strategies can be employed. The same fluorophore attached to different DO sequences can be used to label different RCA products in consecutive cycles of labeling and imaging. Each DO sequence detects just a subset of the RCA products in each cycle. Once the image has been recorded, the fluorescence can be depleted by photo-bleaching [34], or the temperature can be increased so that the DOs dehybridize and can be washed away. The procedure is repeated with a different set of DOs, and a new image can be recorded. With this approach, the number of detected events will grow linearly, based on the number of different fluorophores used in each step and on the number of staining cycles performed [35]. An alternative way to increase the level of multiplexing is to resort to *in situ* sequencing. It relies on the sequential recognition of a four-base DNA sequence tag, and thus the number of different identities detected grows exponentially: theoretically, 4^n^, where four is the number of bases in the tag, and n equals the number of staining cycles [36]. Four cycles can thus detect 256 different unique sequences.

In the living cell, FRET imaging for multiple protein interactions is severely limited by the availability of donor and acceptor fluorophores with suitable spectra. Therefore, going beyond 2–3 different FRET pairs in one cell is virtually impossible. In theory, different donor fluorophores can be distinguished using FLIM (fluorescence-lifetime imaging microscopy); in practice, however, this proves to be extremely challenging (unpublished data).

## 5. It’s Gonna Be a Bright, Bright Sun-Shiny Day

In order to provide more coherent views on the functional states of cells, the level of multiplexing of protein–protein interactions must be increased. Measurement of single analytes is not enough to encompass the complexity of the cell. New tools will shed light on processes that are still in the dark, revealing the beautiful canvas of life. The different technologies described herein all have their advantages and drawbacks, so the choice of method depends on what question needs answering. There is also the possibility of combining the methods described above—for example, to utilize the superior resolution of the new types of microscopy in combination with molecular tools, or to apply different types of molecular tools in conjunction (e.g., *in situ* PLA and FRET) [37]. Microscopic analysis retaining the architectural information of where each analyzed cell is positioned, combined with highly multiplexed information on signaling network activity measured by mRNA-, protein expression, as well as post-translational modifications and protein interactions [38,39] will facilitate studies on cellular communication and on how these signals are interpreted in cells with different genetic and epigenetic background. In addition to the development of improved microscopy and more advanced molecular methods, high-content analysis of tissue sections will require new image analysis and data mining tools in order to provide an output that facilitates the interpretation of the data [40]. Seeing our microcosmos in more and more colors and at a higher and higher resolution will give us an understanding of the organization of both healthy tissues and tissues affected by disease. The future of proteomics will undoubtedly be brighter and more colorful!
